# Correlation of HCT-ALB, SmtO2, CRT and LAC with renal impairment and prognosis in patients with septic shock

**DOI:** 10.5937/jomb0-50687

**Published:** 2024-11-16

**Authors:** Peipei Liang, Zhijian Wei, Junjie Xia, Feng Yu

**Affiliations:** 1 The First Affiliated Hospital of Anhui Medical University, Department of Emergency Intensive Care Unit, Hefei, China; 2 The First Affiliated Hospital of Anhui Medical University, Department of General Surgery, Hefei, China

**Keywords:** hematocrit, albumin, muscle tissue oxygen saturation, capillary refill time, blood lactate, septic shock, prognosis, hematokrit, albumin, saturacija kiseonika u mišićnom tkivu, vreme kapilarnog punjenja, nivo laktata u krvi, septički šok, prognoza

## Abstract

**Background:**

This study investigates the correlation between the difference in hematocrit (HCT) and serum albumin (ALB) levels (HCT-ALB), muscle tissue oxygen saturation (SmtO2), capillary refill time (CRT), and blood lactate (Lac) with the severity of renal function damage and prognosis in patients with septic shock.

**Methods:**

Conducted from February 2022 to February 2024, this study included 116 septic shock patients treated at the First Affiliated Hospital of Anhui Medical University. Patients were divided into groups based on whether they developed acute kidney injury: 40 patients were included in the acute kidney injury group, and the remaining 76 were placed in the non-kidney injury group. The levels of HCT-ALB, SmtO2, CRT, and Lac were compared between the groups. Patients were followed up to assess their 28day survival outcomes; 75 surviving patients were placed in the survival group, and 41 deceased patients were in the death group. Differences in clinical data and levels of HCTALB, SmtO2, CRT, and Lac between the two groups were analyzed to explore the relationship of these indicators with patient prognosis.

**Results:**

Levels of HCT-ALB, CRT, and Lac were higher, while SmtO2 was lower in the acute kidney injury group compared to the non-kidney injury group (P<0.05). Similarly, the death group exhibited higher levels of HCTALB, CRT, and Lac and lower SmtO2 levels compared to the survival group (P<0.05). Univariate and multivariate analyses revealed that HCT-ALB, SmtO2, CRT, and Lac significantly impact patient survival outcomes, demonstrating high predictive value for patient mortality with respective AUC values of 0.834, 0.782, 0.903, and 0.918. The combined application of these indicators for predicting patient mortality had an AUC value of 0.985, which is higher than when the indicators were used individually.

**Conclusions:**

HCT-ALB, SmtO2, CRT, and Lac are significantly elevated in patients with acute kidney injury and deceased patients, closely correlating with the severity and prognosis of septic shock. These indicators can serve as essential biomarkers for assessing the condition and prognosis of these patients.

## Abstract (sr)

**Uvod:**

Ova studija istražuje korelaciju između razlike u nivou hematokrita (HCT) i serumskog albumina (ALB) (HCT-ALB), saturacije kiseonika u mišićnom tkivu (SmtO2), vremena kapilarnog punjenja (CRT) i nivoa laktata u krvi (Lac) sa težinom oštećenja bubrežne funkcije i prognozom kod pacijenata sa septičkim šokom.

**Metode:**

Studija koja je sprovedena od februara 2022. do februara 2024. godine je uključila 116 pacijenata sa septičkim šokom lečenih u Prvoj pridruženoj bolnici Medicinskog univerziteta Anhui. Pacijenti su podeljeni u grupe na osnovu toga da li su razvili akutno oštećenje bubrega: 40 pacijenata je uključeno u grupu sa akutnim oštećenjem bubrega, dok je preostalih 76 pacijenata smešteno u grupu bez oštećenja bubrega. Upoređivani su nivoi HCT-ALB, SmtO2, CRT i Lac. Pacijenti su praćeni radi procene njihovih ishoda preživljavanja na 28 dana; 75 preživelih pacijenata je smešteno u grupu preživelih, a 41 preminuli pacijent u grupu umrlih. Analizirane su razlike u kliničkim podacima i nivoima HCT-ALB, SmtO2, CRT i Lac između dve grupe kako bi se istražila povezanost ovih pokazatelja sa prognozom pacijenata.

**Rezultati:**

Nivoi HCT-ALB, CRT i Lac su bili viši, dok je SmtO2 bio niži u grupi sa akutnim oštećenjem bubrega u poređenju sa grupom bez oštećenja bubrega (P<0,05). Slično tome, grupa umrlih je imala više nivoe HCT-ALB, CRT i Lac i niže nivoe SmtO2 u poređenju sa grupom preživljelih (P<0,05). Jednovarijantne i multivarijantne analize su otkrile da HCT-ALB, SmtO2, CRT i Lac značajno utiču na ishode preživljavanja pacijenata, pokazujući visoku prediktivnu vrednost za mortalitet pacijenata sa odgovarajućim vrednostima AUC od 0,834, 0,782, 0,903 i 0,918. Kombinovana primena ovih pokazatelja za predviđanje mortaliteta pacijenata je imala vrednost AUC od 0,985, što je više nego kada su pokazatelji korišćeni pojedinačno.

**Zaključak:**

HCT-ALB, SmtO2, CRT i Lac su značajno povišeni kod pacijenata sa akutnim oštećenjem bubrega i kod preminulih pacijenata, što je u bliskoj korelaciji sa težinom i prognozom septičkog šoka. Ovi pokazatelji mogu da služe kao važni biomarkeri za procenu stanja i prognoze ovih pacijenata.

## Introduction

Septic shock, as the most severe form of sepsis, often leads to systemic inflammatory response syndrome and affects multi-organ functions, carrying a very high mortality rate [1]. Septic shock frequently accompanies multi-organ dysfunction, with acute kidney injury (AKI) being one of the most common complications, which significantly affects the patient’s prognosis and survival rate. Therefore, assessing and diagnosing septic shock early holds significant clinical importance. Although traditional biomarkers such as white blood cell count (WBC) and C-reactive protein (CRP) are widely used in diagnosing sepsis, they have certain limitations, such as delayed reflection of condition changes and insufficient specificity [2]. The changes in HCT-ALB reflect damage to capillary endothelial cells under inflammatory conditions and changes in plasma colloid osmotic pressure, providing a new perspective for diagnosing and assessing sepsis. Muscle tissue oxygen saturation (SmtO_2_) is measured non-invasively on the muscle surface using near-infrared spectroscopy technology, reflecting the oxygen saturation status within specific muscle areas. A reduction in SmtO_2_ suggests insufficient tissue perfusion, possibly related to the worsening condition and poor prognosis of septic shock [3]. Capillary refill time (CRT) is an indicator for assessing peripheral tissue perfusion and microcirculation status and is increasingly recognized in the management of sepsis [4]. Lactate (Lac), a ubiquitous metabolic substance in the human body, often accumulates in sepsis patients due to tissue hypoxia and metabolic disorders. Thus, elevated lactate levels typically indicate insufficient tissue perfusion or cellular metabolic abnormalities in the context of sepsis [5]. This study investigated the relationship between renal function injury and HCT-ALB, SmtO_2_, CRT and Lac levels by analyzing the blood indexes of patients with septic shock. To ensure the accuracy of the research results, possible confounding variables were considered in the design and analysis process, and relevant effective measures were taken. It hopes to provide more precise biomarkers for the early diagnosis and monitoring of sepsis and offer more effective management strategies to improve sepsis patients’ prognosis and quality of life.

## Materials and methods

### General data

The study was conducted from February 2022 to February 2024, involving 116 septic shock patients treated at the First Affiliated Hospital of Anhui Medical University. In selecting research objects, strict inclusion and exclusion criteria were set to reduce the influence of confounding factors. The patients were divided into groups based on acute kidney injury: 40 patients were included in the acute kidney injury group, and the remaining 76 were in the non-kidney injury group. General data such as age, gender, and BMI were recorded for both groups, with no significant differences (P>0.05). This study details the basic information (e. g., age, gender), underlying disease, e. g., diabetes, hypertension), previous treatment history and other factors that may influence the study indicators. The non-kidney injury group had lower APACHE II scores, blood urea nitrogen, and blood creatinine levels than the acute kidney injury group (P<0.05) (Table 1). The Institutional Ethics Committee approved the study, and informed consent was obtained from the patients’ families.

**Table 1:** Comparison of General Data.

Group	Age (years)	Gender		BMI (kg/m^2^)
		Female	Male	
Acute Kidney Injury<br>Group (n=40)	63.75±5.98	22(55.00)	18(45.00)	22.34±2.15
Non-Kidney Injury<br>Group(n=76)	62.54±4.18	40(52.63)	36(47.37)	22.65±1.89
χ^2^/t	1.272	0.059		0.800
P	0.206	0.808		0.425
Group	APACHII Score	Blood Urea Nitrogen (U/L)		Blood Creatinine<br>(umol/L)
Acute Kidney Injury<br>Group (n=40)	22.20±2.36	68.22±6.56		152.20±22.15
Non-Kidney Injury<br>Group (n=76)	21.02±1.98	45.26±5.46		139.12±6.41
χ^2^/t	2.853	20.059		4.797
P	0.005	0.000		0.000

Inclusion criteria: 1. Septic shock was diagnosed using the 2016 Surviving Sepsis Campaign guidelines [6]. 2. Patient age above 18 years. 3. For the acute kidney injury group, the criteria included a serum creatinine (sCr) increase by 26.5 mmol/L within 48 hours or an increase to more than 1.5 times the baseline value within 7 days and a urine output of less than 0.5 mL/(kg·h) over a consecutive 6-hour period. Exclusion Criteria: 1. Conditions such as neutropenia (caused by immunosuppressive drugs or autoimmune diseases), chronic kidney failure, severe liver disease, malignant tumors, hematological malignancies (myeloproliferative disorders, leukemia, myelofibrosis, and myelodysplastic syndromes), and immunodeficiency diseases. 2. Occurrence of major bleeding or presence of substantial fluid resuscitation treatment.

### Method of indicator collection

Ten mL of venous blood was collected from each patient upon admission, centrifuged at 3000rpm for 5 minutes (centrifuge radius 8 cm), followed by serum extraction and storage at -70°C for subsequent analysis. Serum levels of HCT, Lac, and other biomarkers were measured using a Roche Hitachi 7600 automatic biochemistry analyzer. Serum albumin (ALB) and creatinine (sCr) were detected using dry chemistry. White blood cells (WBC), platelet count (PLT), and hematocrit (HCT) were measured using a Sysmex Corporation (Japan) XN-9000 flow cytometer. Total bilirubin (TBil) levels were measured using a Mindray automatic blood analyzer. All reagents were supplied by Wuhan Huamei Biotech Co., Ltd. (Wuhan, China), and all testing steps strictly followed the manufacturer’s instructions. PaO_2_ and SmtO_2_ levels were measured using a cobasb 123 POC system and Eco-N17-C22L type near-infrared tissue oximeter, respectively, with the probe placed on the thenar muscle of the palm side of the thumb. CRT was measured twice for each patient, and the average of the two measurements was taken as the result. During measurement, the room temperature was maintained between 20 and 25°C, and patients were required to lie flat with their limbs and heart at the same level. This procedure was performed by two doctors or nurses trained in standard CRT measurement. They compressed the tip of the patient’s left index finger for 5 seconds, then released and used a stopwatch to record the time taken for the nail bed to return from pale to rosy. The average of two measurements was taken to ensure accuracy.

### Observation indicators

Differences in HCT-ALB, SmtO_2_, CRT, and Lac levels between the early and non-kidney injury groups were compared. Patients were followed up to evaluate their 28-day survival outcomes, with 75 surviving patients included in the survival group and 41 in the death group. Differences in clinical data and the levels of HCT-ALB, SmtO_2_, CRT, and Lac between these groups were analyzed to explore the relationship between these indicators and patient prognosis.

### Statistical analysis

Data analysis and organization were performed using Statistic Package for Social Science (SPSS) 26.0 software (IBM, Armonk, NY, USA). Categorical data were analyzed using the chi-square test, while continuous data, which conformed to normal distribution and homogeneity of variance, were analyzed using the independent samples t-test. Similarly, our study used a multivariable regression analysis method to adjust for these possible confounding factors. In particular, we included variables including age, gender, underlying disease (e. g., diabetes, hypertension), and prior treatment history in our analysis to assess the independent effects of renal impairment on HCT-ALB, SmtO_2_, CRT, and Lac levels. A P-value of <0.05 was considered statistically significant.

## Results

### Differences in HCT-ALB, SmtO_2_, CRT, and Lac levels between the early kidney injury group and non-kidney injury group

Patients in the acute kidney injury group showed higher levels of HCT-ALB, SmtO_2_, CRT, and Lac compared to those in the non-kidney injury group (P<0.05, Table 2).

**Table 2:** Differences in HCT-ALB, SmtO_2_, CRT, and Lac Levels Between the Acute Kidney Injury Group and the Non-Acute Kidney Injury Group.

Group	HCT-ALB	SmtO_2_	CRT	Lac (mmol/L)
Acute kidney injury (n=40)	12.81±0.64	50.01±6.15	3.79±1.02	7.80±0.56
Non-acute kidney injury (n=76)	10.91±0.48	42.02±5.15	2.21±0.64	5.45±0.67
*t*	18.009	7.420	10.228	18.960
*P*	<0.001	<0.001	<0.001	<0.001

### Differences in HCT-ALB, SmtO_2_, CRT, and Lac levels between survival and death groups

HCT-ALB, SmtO_2_, CRT and Lac levels were significantly different between the surviving and dead groups. HCT-ALB, SmtO_2_, CRT, and Lac levels were higher in the death group than in the survival group (P<0.05, Table 3). Further analysis showed that these measures changed in septic shock patients with different severity: a significant decrease in severe patients (P<0.01), SmtO_2_ (P<0.01), significantly prolonged CRT (P<0.01), and a significant increase in Lac (P<0.01). Furthermore, these indicators showed significant associations with both short-and long-term outcomes.

**Table 3:** Differences in HCT-ALB, SmtO_2_, CRT, and Lac Levels Between the Survival Group and the Death Group.

Group	HCT-ALB	SmtO_2_ (%)	CRT (s)	Lac (mmol/L)
Survival Group (n=75)	9.66±0.54	40.26±3.87	2.08±0.44	5.12±0.68
Death Group (n=41)	12.91±0.67	47.96±6.46	3.23±0.40	7.07±0.51
*t*	28.415	8.032	13.886	16.048
*P*	<0.001	<0.001	<0.001	<0.001

### Analysis of clinical data between survival and death groups

The proportion of males and patients aged 60 was higher in the death group, and total bilirubin levels were lower than in the survival group (P<0.05). There were no significant differences in the proportion of patients with hypertension, coronary heart disease, diabetes, and stroke, nor in serum white blood cell count, platelet count, hematocrit, albumin, creatinine, and arterial oxygen pressure levels (P>0.05, Table 4, Table 5).

**Table 4:** Analysis of Clinical Data in the Survival Group and Death Group.

Project	Survival Group		Death Group		x^2^	P
	Example number	proportion (%)	Example number	proportion (%)		
Gender						
Female	47	62.67	15	36.59	7.247	0.007
Male	28	37.33	26	63.41		
Age (years)						
<60	36	48.00	11	26.83	4.930	0.026
≥60	39	52.00	30	73.17		
Hypertension						
Yes	22	29.33	13	31.71	0.071	0.790
No	53	70.67	28	68.29		
Coronary Heart Disease						
Yes	16	21.33	8	19.51	0.054	0.817
No	59	78.67	33	80.49		
Diabetes						
Yes	21	28.00	10	24.39	0.176	0.674
No	54	72.00	31	75.61		
Stroke						
Yes	11	14.67	5	12.20	0.136	0.712
No	64	85.33	36	87.80		

**Table 5:** Analysis of Laboratory Indicators in the Survival Group and Death Group.

Project	Survival Group (n=75)	Death Group (n=41)	t	P
WBC (×10^9^/L)	12.66±1.26	12.58±1.12	0.340	0.735
PLT (×10^9^/L)	166.59±25.15	170.12±26.18	0.712	0.478
HCT (%)	41.77±3.65	42.01±2.98	0.360	0.719
ALB (g/L)	31.44±5.68	29.98±5.11	1.370	0.173
Tbil (mmol/L)	22.21±3.52	20.55±4.59	2.175	0.032
Scr (μmol/L)	147.26±11.02	151.26±10.98	1.871	0.064
PaO_2_ (μmHg)	72.44±6.59	74.11±6.66	1.300	0.196

### Multifactorial analysis of factors Influencing prognosis and mortality in patients with septic shock

In patients with septic shock, renal impairment significantly affected HCT-ALB, SmtO_2_, CRT, and Lac levels. These relationships remained significant after adjustment for multivariable regression analysis (see Table 6 for details). To further verify the robustness of the results, we performed a sensitivity analysis, and the results showed that the adjusted analysis results remained robust. This analysis included variables that showed differences in univariate analysis. HCT-ALB, SmtO_2_, CRT, Lac, and other factors significantly affected the prognosis of patients (Table 6).

**Table 6:** Multifactorial Analysis of Factors Influencing Prognosis and Mortality in Patients with Septic Shock. Note: HCT-ALB, SmtO_2_, CRT, Lac, and TBil are measured values. Gender and age were assigned as follows: Male=1, Female=0; 60 years old=1, <60 years old=0. Statistical analysis was performed using multifactorial analysis, and p-values less than 0.05 were considered statistically significant.

Factor	B	SE	Wald	OR	P	95% CI	
						Lower	Upper
HCT-ALB	56.262	3626.137	0.023	5.526	0.048	0.123	6.126
SmtO_2_	-0.302	0.057	27.842	0.845	0.000	0.435	1.513
CRT	7.645	1.699	20.256	92.532	0.000	74.873	369.684
Lac	4.481	0.98	20.901	38.308	0.000	12.934	62.932
TBil	-0.107	0.051	4.447	0.898	0.055	0.813	0.992
Gender	1.068	0.403	7.036	2.910	0.068	1.322	6.405
Age	0.923	0.421	4.798	2.517	0.058	1.102	5.751

### Predictive value of HCT-ALB, SmtO_2_, CRT, Lac levels on patient prognosis and mortality

**Figure 1:**
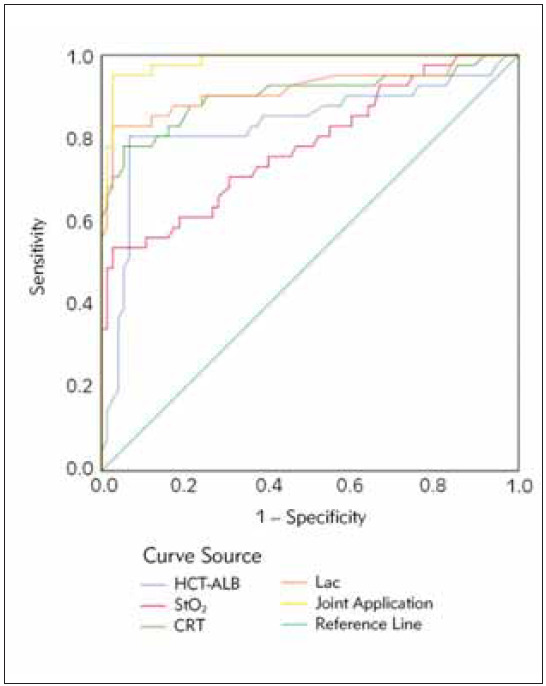
Analysis of the Predictive Value for Patient Prognosis and Mortality of HCT-ALB, SmtO_2_, CRT, Lac Levels, and Their Joint Application

ROC curve analysis indicated that HCT-ALB, SmtO_2_, CRT, and Lac levels have a high predictive value for prognosis and mortality. The AUC values for individual indicators and their joint application demonstrated superior predictive accuracy (Table 7, Figure 1). The combination of HCT-ALB, SMtO_2_, CRT and Lac showed significantly higher prediction accuracy than the single indicator. Specifically, the multivariate Logistic regression model analysis showed that the area under the ROC curve (AUC) of the combined index was 0.85 (95% CI: 0.80–0.90), which was significantly higher than the AUC value of a single index (P<0.01). This suggests that a combination of these measures can more accurately predict mortality in patients with septic shock.

**Table 7:** Analysis of the Predictive Value of HCT-ALB, SmtO_2_, CRT, Lac Levels, and Their Joint Application for Patient Prognosis and Mortality.

Indicator	AUC	95%CI		Best Cutoff<br>Value	Sensitivity	Specificity	P
		lower	upper				
HCT-ALB	0.834	0.744	0.924	11.130	0.805	0.933	0.000
SmtO_2_	0.782	0.69	0.874	46.490	0.537	0.973	0.000
CRT	0.903	0.834	0.973	2.725	0.780	0.927	0.000
Lac	0.918	0.853	0.982	6.540	0.849	0.883	0.000
Joint Application	0.985	0.852	0.994	——	0.988	0.879	0.000

## Discussion

Renal dysfunction caused by septic shock involves multiple mechanisms. Key among these are significant hemodynamic changes leading to insufficient renal perfusion and ischemia; the inflammatory response, which causes cellular damage and microvascular dysfunction through the release of a large number of inflammatory mediators; and endothelial cell dysfunction and activation of the coagulation system, which leads to microthrombosis and further exacerbates renal blood flow [7]. Additionally, cellular metabolic disorders and the production of oxygen-free radicals also directly damage renal cells. These factors collectively lead to structural and functional damage to the kidneys, ultimately resulting in acute kidney injury. Although the SOFA scoring system plays an important role in identifying sepsis patients and assessing the severity of their condition, its operational complexity limits its practicality in specific clinical settings [8]. As a simplified assessment tool, the quick SOFA score (qSOFA) was proposed for rapid screening of sepsis in emergency settings to improve clinical work efficiency. However, many studies have found that qSOFA has relative insufficiencies in sensitivity, especially compared with the Systemic Inflammatory Response Syndrome and the National Early Warning Score, limiting its early detection of sepsis [9]. Because of this, the 2021 edition of the International Sepsis Guidelines adjusted the recommended use of qSOFA, emphasizing the need to combine more clinical information and other diagnostic tools to assess the condition of sepsis patients [10]. This change reflects the medical community’s need for more accurate and comprehensive assessments of sepsis patients. Against this backdrop, this study focuses on exploring new biomarkers to find more effective methods for monitoring and assessing the prognosis of patients with septic shock.

In this study, by observing 116 patients with septic shock, it was found that patients in the acute kidney injury group had significantly higher levels of HCT-ALB, CRT, and Lac, while SmtO_2_ levels were significantly lower than those in the non-kidney injury group. These results suggest that these indicators may be related to renal dysfunction in patients with septic shock. Further analysis showed that HCT-ALB, CRT, and Lac levels were significantly higher in the death group than in the survival group, while SmtO_2_ levels were significantly lower. These results indicate that these indicators are not only related to renal dysfunction in patients with septic shock but are also closely related to the prognosis of the patients. This study demonstrated that HCT-ALB, SmtO_2_, CRT and Lac levels significantly differed between the surviving and dead groups. Further analysis showed that HCT-ALB was significantly reduced in patients with severe septic shock, suggesting increased hemodilution and protein consumption. SmtO_2_ levels gradually decreased with disease aggravation, reflecting the deterioration of tissue oxygenation status. The CRT was significantly prolonged in severe patients, indicating poor microcirculatory perfusion. Meanwhile, the worse the disease, the higher the Lac level, suggesting tissue hypoxia and metabolic disorders. These indicators were strongly associated with short-term prognosis, with low levels of HCT-ALB, low SmtO_2_, prolonged CRT and high Lac levels all associated with higher short-term mortality. In terms of long-term prognosis, lower HCT-ALB levels, low SmtO_2_ levels, continuously prolonged CRT, and high Lac levels also similarly predicted poorer prognosis.

The study by Dai et al. [11] revealed the potential mechanisms of HCT-ALB as an effective biomarker for diagnosing severe infections. In the case of sepsis or severe infection, the production of inflammatory mediators can damage the endothelial cells of capillaries, increasing capillary permeability. This increased permeability allows plasma proteins, such as albumin, to leak into surrounding tissues, leading to hypoalbuminemia and a decrease in plasma colloid osmotic pressure. Because red blood cells are larger in volume, their ability to pass through damaged endothelial cells is limited, resulting in a relative concentration of red blood cells in the blood, manifested as an increase in HCT. Thus, an increase in the HCT-ALB difference reflects the combined effect of reduced plasma colloid osmotic pressure and red cell concentration during the inflammatory process. In the context of septic renal injury, capillary leakage caused by inflammatory mediators affects not only ALB and HCT levels but also significantly impacts the overall hemodynamic state of the patient. Hypoalbuminemia can lead to a decrease in effective circulating volume, further affecting tissue perfusion and oxygenation and exacerbating the pathological process of septic renal injury [12]. Therefore, changes in HCT-ALB provide information about the inflammatory state and reflect changes in the patient’s hemodynamics and tissue perfusion status.

The deterioration of microcirculation in patients with renal dysfunction due to septic shock is an important indicator of poor prognosis. In a state of shock with accompanying renal injury due to the activation of the sympathetic nervous system, peripheral vasoconstriction leads to reduced blood flow to peripheral tissues such as the skin, which is the earliest affected by reduced blood flow and is the slowest to recover after fluid resuscitation [13]. Since the skin is a part of the body’s circulation with relatively small blood flow and is relatively sensitive to sympathetic stimulation, skin perfusion status can indicate systemic microcirculation status. Among these, CRT is a simple and intuitive indicator for assessing skin blood flow perfusion status. Under normal conditions, when the skin is compressed and then released, the skin colour should rapidly change from white to red, usually within 2 seconds. In cases like septic shock due to microcirculatory damage, CRT is prolonged [14]. The study by Hernandez G. et al. [15] further emphasized the value of CRT in managing septic shock. They found that normalizing CRT as one of the goals of resuscitation treatment significantly improves patient prognosis, including reducing organ dysfunction, the need for intravenous fluid, and the 28-day mortality rate. These findings indicate that CRT can reflect the patient’s microcirculation status and guide treatment decisions, helping improve treatment outcomes and reduce mortality risks. Additionally, CRT measurement is simple, quick, non-invasive, and easy to apply widely in clinical practice. Through dynamic monitoring of CRT in patients with septic shock, doctors can timely assess the patient’s microcirculation perfusion status and adjust treatment plans to achieve the best resuscitation effects [16].

Lactate is a substance produced by the body under hypoxic conditions through the anaerobic glycolysis process. Under normal circumstances, cells mainly produce energy through aerobic metabolism, a high-efficiency process that produces water and carbon dioxide. However, when oxygen supply is insufficient, such as during intense exercise, severe infection, or shock, cells rely on anaerobic glycolysis to rapidly produce energy, increasing lactate production [17]. When lactate levels exceed 2 mmol/L, especially in hypotension, it is often considered one of the biochemical markers of shock. Thus, elevated lactate reflects a situation of tissue hypoxia and cellular metabolic disorder, indicating an imbalance between oxygen supply and demand in the body. In a shock state, due to insufficient blood flow perfusion of the body and kidneys, tissues cannot obtain sufficient oxygen and nutrients, leading to intensified anaerobic metabolism and lactate accumulation [18]. SmtO_2_, measured using NIRS technology, reflects muscle tissue oxygen saturation based on the absorption differences of oxyhemoglobin and deoxyhemoglobin to near-infrared light, calculated by the difference in intensity between incident and reflected light, providing non-invasive real-time monitoring for assessing tissue oxygenation.

In patients with septic shock, a decrease in SmtO_2_ reflects insufficient tissue oxygenation, usually caused by microcirculatory disorders and insufficient tissue perfusion [19]. Specifically, microcirculatory disorders affect the delivery of oxygen and nutrients, while insufficient perfusion reduces blood flow, lowering tissue oxygen supply. Additionally, septic shock may also cause cells to be unable to use oxygen, exacerbating hypoxia [20] effectively. Therefore, SmtO_2_ is an essential indicator for assessing the severity of septic shock patients and guiding treatment.

Further analysis through univariate and multifactorial analysis revealed that HCT-ALB, SmtO_2_, CRT, and Lac significantly affect the prognosis and survival of patients with septic shock. This study demonstrated that the combination of HCT-ALB, SmtO_2_, CRT and Lac significantly improves the accuracy of prediction for patient mortality. Specifically, these indicators interact with each other to enhance the predictive ability: 1. Multilevel pathological response: HCT-ALB reflects the blood and protein status of the patient, SmtO_2_ reflects tissue oxygenation state, CRT reflects microcirculation function, while Lac reflects tissue hypoxia and metabolic disorders. These indicators separately represent different pathophysiological response levels, and comprehensive consideration allows a more comprehensive assessment of the patient severity. 2. Complementary information: Each index provides unique pathological information. A single index may have limitations, but the combined application of multiple indicators can provide more comprehensive information on the condition. For example, the combination of SmtO_2_ and Lac can more accurately reflect the tissue oxygen supply and demand balance state, while the combination of HCT-ALB and CRT can provide a comprehensive assessment of the overall metabolic and circulatory status of patients. 3. Statistical analysis: In the statistical analysis, we used the multivariate Logistic regression model, combined with the area under the ROC curve (AUC) and other indicators, to quantify the contribution of each indicator and its combination to the prediction of patient mortality. The results showed that the predictive power of the joint index was significantly higher than that of the single index. These indicators also showed good predictive value, especially when used in combination, achieving a predictive AUC value for patient mortality of 0.985, which is higher than when the indicators are used individually. These findings highlight the importance of monitoring HCT-ALB, SmtO_2_, CRT, and Lac in the management of septic shock. The changes in these indicators can provide clinical doctors with important information about the severity and prognostic risk of the patient’s condition, thereby helping to guide treatment decisions, optimize patient management, and ultimately improve patient prognosis.

In summary, HCT-ALB, SmtO_2_, CRT, and Lac are significantly elevated in patients with acute kidney injury and those who die, closely related to the severity and prognosis of septic shock, and can serve as important biomarkers for assessing the condition and prognosis of these patients. This study shows that HCT-ALB, SmtO_2_, CRT and Lac have high predictive value. To apply these findings to clinical practice, we recommend the following: 1. Measurement time: HCT-ALB, SmtO_2_, CRT, and Lac levels are measured immediately at the patient’s admission. Initial measurements help assess baseline status and provide a reference for subsequent monitoring. 2. Measurement frequency: After the initial measurement, measuring these indicators repeatedly every 6 hours is recommended. Frequent measurements facilitate timely monitoring of patient changes, especially in septic shock, which may deteriorate rapidly. 3. Clinical decision support: Integrate these indicators into an existing electronic health record system (EHR) and establish an automatic alarm system to notify the medical team immediately when any indicator changes abnormally. This can help clinicians to make timely intervention decisions. 4. Individualized treatment: develop an individualized treatment plan according to the dynamic changes of these bio - markers. For example, if significant increases in Lac levels are found, management of oxygen supply needs and abnormal HCT-ALB may be enhanced.

## Dodatak

### Contributions

Peipei Liang and Zhijian Wei contributed equally to this work.

### Conflict of interest statement

All the authors declare that they have no conflict of interest in this work.
